# Peak early‐phase enhancement ratio on contrast‐enhanced MRI to differentiate chromophobe renal cell carcinoma from oncocytoma

**DOI:** 10.1002/bco2.70017

**Published:** 2025-04-12

**Authors:** Deanna Thorson, Davide Bova, Maria M. Picken, Marcus L. Quek, Gopal N. Gupta, Hiten D. Patel

**Affiliations:** ^1^ Department of Radiology, Feinberg School of Medicine Northwestern University Chicago IL USA; ^2^ Department of Radiology Loyola University Medical Center Maywood IL USA; ^3^ Department of Pathology Loyola University Medical Center Maywood IL USA; ^4^ Department of Urology Loyola University Medical Center Maywood IL USA; ^5^ Department of Surgery Loyola University Medical Center Maywood IL USA; ^6^ Department of Urology, Feinberg School of Medicine Northwestern University Chicago IL USA; ^7^ Surgery Service Jesse Brown VA Medical Center Chicago IL USA

**Keywords:** chromophobe renal cell carcinoma, kidney cancer, magnetic resonance imaging, Oncocytoma, renal cell carcinoma

## Abstract

**Objectives:**

To evaluate the feasibility of using the peak early‐phase enhancement ratio (PEER) of tumour to renal cortex measured on contrast‐enhanced magnetic resonance imaging (MRI) to distinguish between chromophobe renal cell carcinoma (chRCC) and oncocytoma, which are difficult to differentiate on renal mass biopsy.

**Patients and Methods:**

A consecutive case–control study was conducted of patients with chRCC or oncocytoma based on surgical pathology (2006–2020). Two radiologists blinded to pathology results independently measured PEER values on MRI for each tumour. PEER values were compared with surgical pathology results.

**Results:**

For the 18 renal tumours evaluated, PEER values were higher for the 7 oncocytomas than for the 11 chRCCs (median 1.33 versus 0.55, p < 0.001). Agreement between the image interpreters was high (Pearson's: 0.90). PEER cutoff values ranging from 0.98 to 1.05 provided high performance in identifying chRCC. A PEER cutoff value of ≤1.05 had sensitivity, specificity, positive predictive value (PPV) and negative predictive value (NPV) of 100% for the averaged PEER measurements between the two radiologists. High accuracy in identifying chRCC was also achieved for each individual image interpreter using the cutoff value of ≤1.05, with sensitivity of 100%, specificity of 85.7%, PPV of 91.7% and NPV of 100% for radiologist #1 and sensitivity of 90.9%, specificity of 85.7%, PPV of 90.9% and NPV of 85.7% for radiologist #2.

**Conclusion:**

Differentiating chRCCs from oncocytomas using PEER measurements obtained from contrast‐enhanced MRI is feasible and reproducible between radiologists. We identified an accurate range for PEER cutoff values (0.98 to 1.05) requiring validation and adjustment in additional cohorts to maintain high sensitivity for detecting chRCC and negative predictive value. Using MRI PEER to evaluate oncocytic tumours with a differential diagnosis of chRCC versus oncocytoma based on biopsy pathology may help avoid unnecessary intervention for oncocytomas.

## 1 INTRODUCTION

Renal cell carcinoma (RCC) incidence has increased over the past several decades, at least in part due to the increased use of cross‐sectional imaging. The stability in mortality accompanying the increased incidence of RCC suggests overdiagnosis and raises concern for overtreatment.^1^ Of the >60 000 RCCs diagnosed annually in the United States, the majority present as a renal mass without distant disease.^2^ These malignant masses are difficult to distinguish from benign solid renal masses based on imaging or clinical features, and an estimated 20–30% of resected renal masses are benign depending on size.^3^, ^4^

The majority of benign resected renal masses are oncocytomas, which are not readily distinguished from RCC on conventional imaging evaluation.^5^, ^6^ Percutaneous biopsy can be helpful in establishing a diagnosis if certain RCC subtypes are detected, however, >30% of benign biopsy results may be false negatives based on tumours selected for resection.^7^ Accuracy in distinguishing benign oncocytoma from the chromophobe subtype of RCC (chRCC) on biopsy is particularly limited because these tumours share many histologic features. Specifically, positive tumour staining for protein biomarker CD117 differentiates these tumours from other RCC subtypes but is seen with both chRCC and oncocytoma. The positive predictive value for distinguishing benign oncocytoma from chRCC on biopsy is reported as low as 67%, indicating that up to a third of tumours presumed to be benign oncocytoma on biopsy are ultimately found to be RCC upon surgical excision.^8^ Given the challenges in histopathologic differentiation of oncocytoma from chRCC especially on limited biopsy samples, these tumour types are often reported as “oncocytic neoplasm” on renal mass biopsy. This result indicates that chRCC and benign oncocytoma both remain in the differential and therefore may be insufficient for guiding appropriate management.

A recently described method measuring the peak early‐phase enhancement ratio (PEER) between tumour and renal cortex on computed tomography (CT) examinations reported up to 100% accuracy in differentiating CD117 positive benign oncocytomas from chRCC.^9^ An additional study externally validating this CT PEER measurement method found a similarly high sensitivity of 93% for distinguishing chRCC from oncocytoma regardless of tumour CD117 status on pathology evaluation and a sensitivity of 97% for identifying chRCC in the CD117 positive tumour subset.^10^ However, renal masses may alternatively be evaluated with magnetic resonance imaging (MRI) rather than CT due to advantages in contrast safety for patients with decreased renal function and in lesion characterization (e.g. identifying hemorrhagic or intracellular fat components), resulting in potentially greater specificity for imaging diagnosis of RCC.^11^ Therefore, we aimed to evaluate whether it is feasible to use PEER measurement on contrast‐enhanced MRI to differentiate chRCC from oncocytoma. Applying PEER assessment to renal masses with pathology of oncocytic neoplasm on percutaneous biopsy (implying a differential diagnosis of chRCC versus oncocytoma) could facilitate confident differentiation between chRCC which may require resection and oncocytomas which can be managed with surveillance, thus reducing the rate of unnecessary nephrectomies for benign oncocytomas.

## 2 PATIENTS AND METHODS

### 2.1 Patient selection

Internal review board approval was obtained for this single institution, diagnostic retrospective consecutive case–control study. Institutional pathology reports were searched utilizing CoPath natural language search tool to identify all patients who had undergone radical or partial nephrectomy with final surgical pathology demonstrating oncocytoma or chRCC regardless of CD117 status from 2006 to 2020. Patients without any preoperative imaging available for review were excluded. Patients with greater than 2 concurrently present solid renal masses on imaging were excluded, and patients with 2 masses without pathology available for each tumour were excluded. Patients who had undergone a presurgical pre‐ and post‐contrast MRI examination of the kidneys with images available for review were included for analysis in the study.

### 2.2 Image acquisition

The MRI examinations were performed on multiple commercially available 1.5 Tesla and 3 Tesla closed‐bore MRI units: 1.5 T Aera, Espree and Sola (Siemens Healthineers, Erlangen, Germany); 3 T Trio and Verio (Siemens); 1.5 T Signa Artist and Signa Optima 450 W (GE Healthcare, Milwaukee, WI, USA); and 3 T Signa Premier (GE) MRI scanners. The dedicated renal examination protocol included T1‐weighted fat‐suppressed images acquired prior to, 30 seconds after, and 90 seconds after intravenous gadolinium‐based contrast administration, corresponding to pre‐contrast, corticomedullary (arterial) and nephrographic (venous) phases, respectively. Some variation in utilized protocols and techniques may have resulted in variations in post‐contrast acquisition timing to approximately 20 and 70 seconds after contrast administration for the arterial and venous phases, respectively.

### 2.3 Image analysis

Two radiologists independently reviewed the preoperative MRI images and made the measurements needed to calculate PEER values for the tumours. Radiologist #1 was a radiology resident, and radiologist #2 was an attending radiologist with expertise in genitourinary imaging and over 20 years of experience in abdominal imaging interpretation. The difference in radiology reviewer experience was intended to evaluate the reproducibility of measurements between radiologists with different levels of expertise. The radiology reviewers were blinded to the pathology results by an independent party.

PEER values were calculated from the MRI images using a technique mirroring the PEER calculation technique for CT which has been previously described in the literature.^9^, ^10^ To measure peak early enhancement within the tumour, the area of highest signal within the tumour on corticomedullary phase post‐contrast T1‐weighted fat‐suppressed images was identified by visual inspection. A single 1.0 cm diameter circular two‐dimensional region of interest (ROI) was placed on that highest signal area of the tumour on the axial corticomedullary phase image, and the average signal intensity measurement within the ROI was recorded. For small, heterogeneous tumours a 1.0 × 0.5 cm elliptical ROI could instead be utilized at the radiologist's discretion to measure the brightest portion of the tumour, in order to avoid measurement of hypoenhancing areas. An ROI of the same size and in the same location within the tumour was then used to measure signal intensity in the tumour on the corresponding pre‐contrast axial T1‐weighted fat‐suppressed image.

**FIGURE 1:**
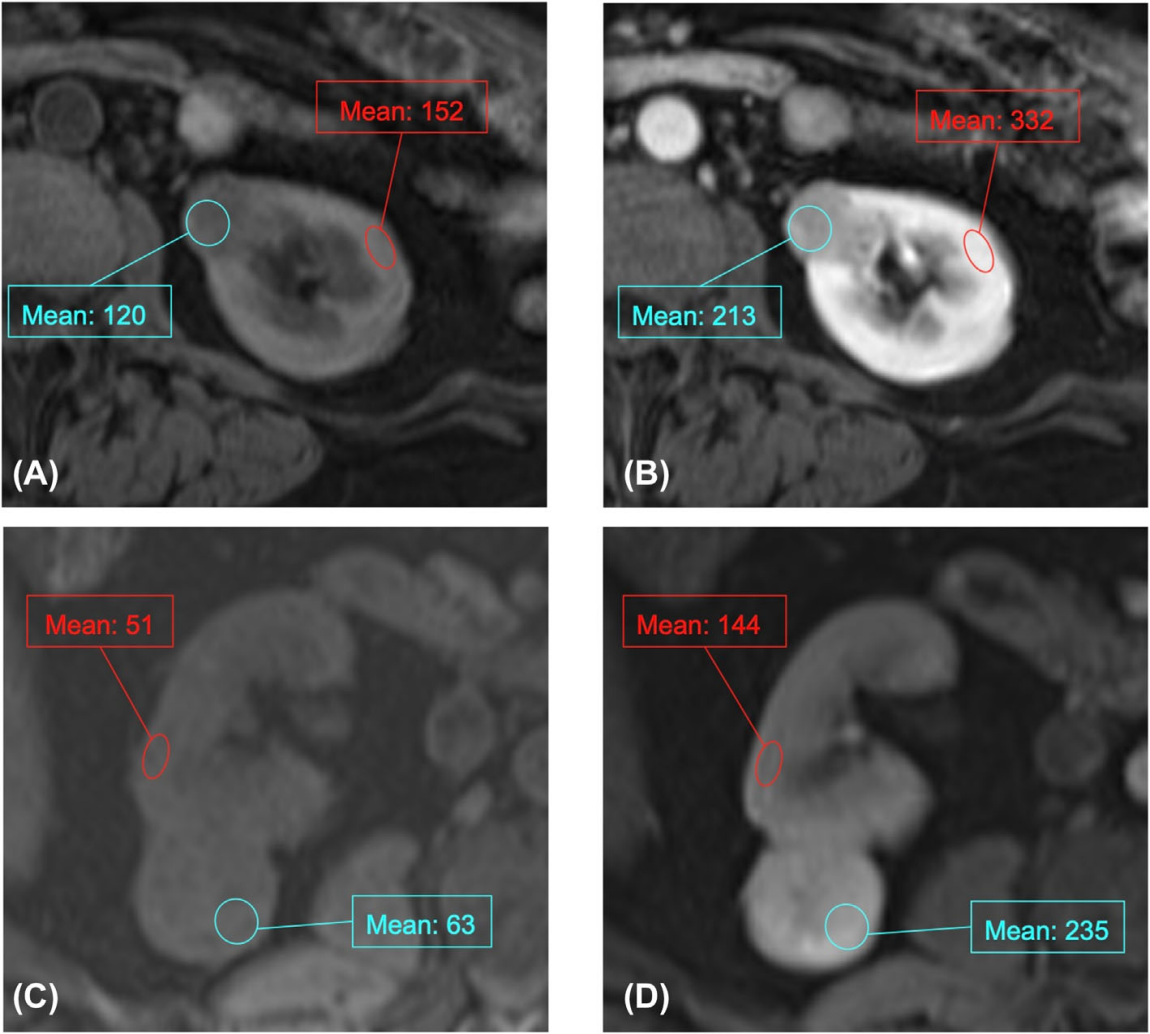
PEER measurement technique. T1‐weighted fat‐suppressed magnetic resonance images demonstrating ROI placement to measure signal intensities for PEER calculation within the tumour (blue circles) and within the renal cortex (red ovals) on (a) pre‐contrast image and (b) post‐contrast image in a patient with chRCC, and (c) pre‐contrast image and (d) post‐contrast image in a patient with oncocytoma. (a, b) PEER = (213–120)/(332–152) = 0.52. (c, d) PEER = (235–63)/(144–51) = 1.85.

To measure enhancement in the renal cortex, an ellipsoid ROI without specified dimensions was placed within a representative portion of the renal cortex on the same image slice as the tumour ROI, in the same location on both the corticomedullary phase post‐contrast and the pre‐contrast T1‐weighted fat‐suppressed images. The PEER value was calculated by dividing the enhancement of the tumour by the enhancement of the cortex. If the corticomedullary phase was absent or of inadequate quality for evaluation, the nephrographic phase was instead used for post‐contrast measurements of the tumour and renal cortex, as per the previously described technique for measuring PEER on CT.^9^ The technique for drawing ROIs and calculating the PEER values is shown in Figure 1.

### 2.4 Pathology review

Pathology re‐review was performed by an expert in genitourinary pathology as needed to definitively characterize resected lesions as chRCC or oncocytoma and determine CD117 status when feasible.

### 2.5 Statistical analysis

The primary outcome analysed was a diagnosis of oncocytoma or chRCC on surgical pathology. Patient clinical characteristics were evaluated using Student's t‐test for continuous parameters and chi‐squared test for categorical parameters. The PEER values from each radiologist and the averaged PEER values between the two radiologists were evaluated for an optimal cutoff for the prediction of oncocytoma versus chRCC on pathology. Sensitivity, specificity, positive predictive value (PPV) and negative predictive value (NPV) were calculated for a range of PEER cutoff values selected based on the distribution of PEER measurements. Receiver operating characteristic (ROC) curve calculations were used to determine the area under the ROC curve (AUC).

## 3 RESULTS

**FIGURE 2:**
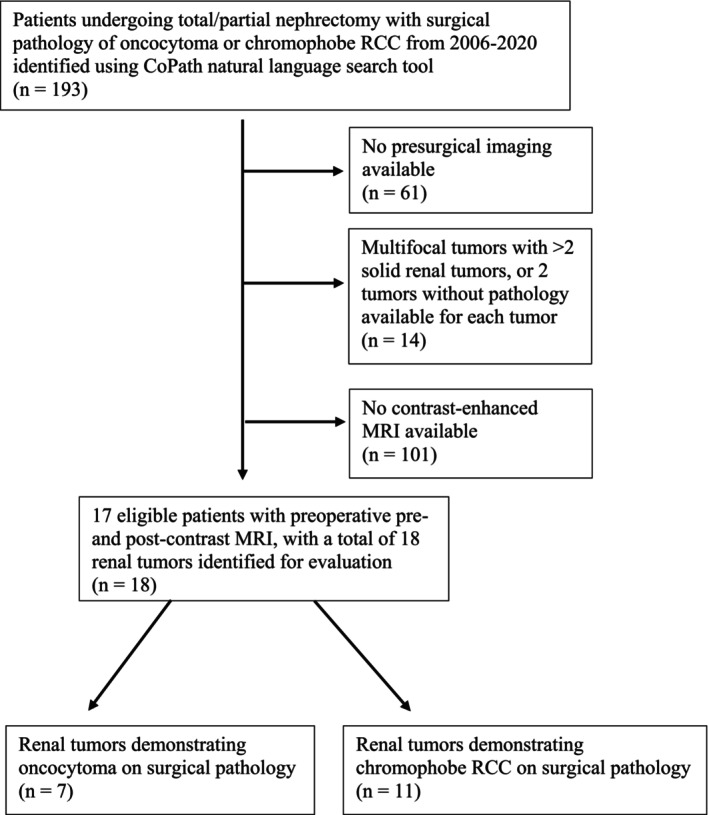
Flowchart demonstrating patient selection.

A total of 17 patients with 18 renal tumours demonstrating surgical pathology of oncocytoma or chRCC with preoperative contrast‐enhanced MRI examinations were identified (Figure 2). Median tumour size was 3.5 cm based on MRI and pathologic measurements. Seven tumours were oncocytomas, while 11 tumours were chRCCs (Table 1). CD117 status was positive for 10/18 (55.6%) and unknown for 8/18 (38.9%) of the tumours. None of the tumours evaluated had negative CD117 status. Two of the tumours analysed did not have adequate corticomedullary phase post‐contrast images available and had PEER measurements performed using the nephrographic phase images; the rest of the tumours were evaluated on corticomedullary phase imaging. All tumours were included for primary analysis based on inclusion criteria.

**TABLE 1:** Baseline demographics and clinical characteristics stratified by the presence of oncocytoma or chromophobe RCC at surgical pathology.

	Overall	Oncocytoma	Chromophobe RCC	p
**Patients (n)**	17	6	11	
**Median age, yr (IQR)**	59.3 (46.9–65.5)	60.2(55.9–82.2)	59.1 (45.0–65.5)	0.50
**Sex, n (%)**				
**Female**	7 (41.2)	2 (33.3)	5 (45.5)	0.63
**Male**	10 (58.8)	4 (66.7)	6 (54.5)	
**Masses (n)**	18	7	11	
**Surgical Approach**				
**Open**	4 (22.2)	3 (42.9)	1 (9.1)	0.14
**Laparoscopic**	4 (22.2)	2 (28.6)	2 (18.2)	
**Robotic**	10 (55.6)	2 (28.6)	8 (72.7)	
**Surgery Type**				
**Radical Nephrectomy**	11 (61.1)	4 (57.1)	7 (63.6)	0.78
**Partial Nephrectomy**	7 (38.9)	3 (42.9)	4 (36.4)	
**Laterality**				
**Left**	7 (38.9)	3 (42.9)	4 (36.4)	0.78
**Right**	11 (61.1)	4 (57.1)	7 (63.6)	
**Median radiographic size, cm (IQR)**	3.5 (2.2–6.8)	2.4 (2.0–3.5)	6.4 (2.2–8.9)	0.04
**Median pathologic size, cm (IQR)**	3.5 (2.4–7.1)	2.6 (2.0–4.5)	6.1 (2.5–9.0)	0.10
**CD117 Status, n (%)**				
**Negative**	0 (0)	0 (0)	0 (0.0)	0.01
**Positive**	10 (55.6)	1 (14.3)	9 (81.8)	
**Unknown**	8 (44.4)	6 (85.7)	2 (18.2)	

Abbreviations: IQR, interquartile range; RCC, renal cell carcinoma.

**FIGURE 3:**
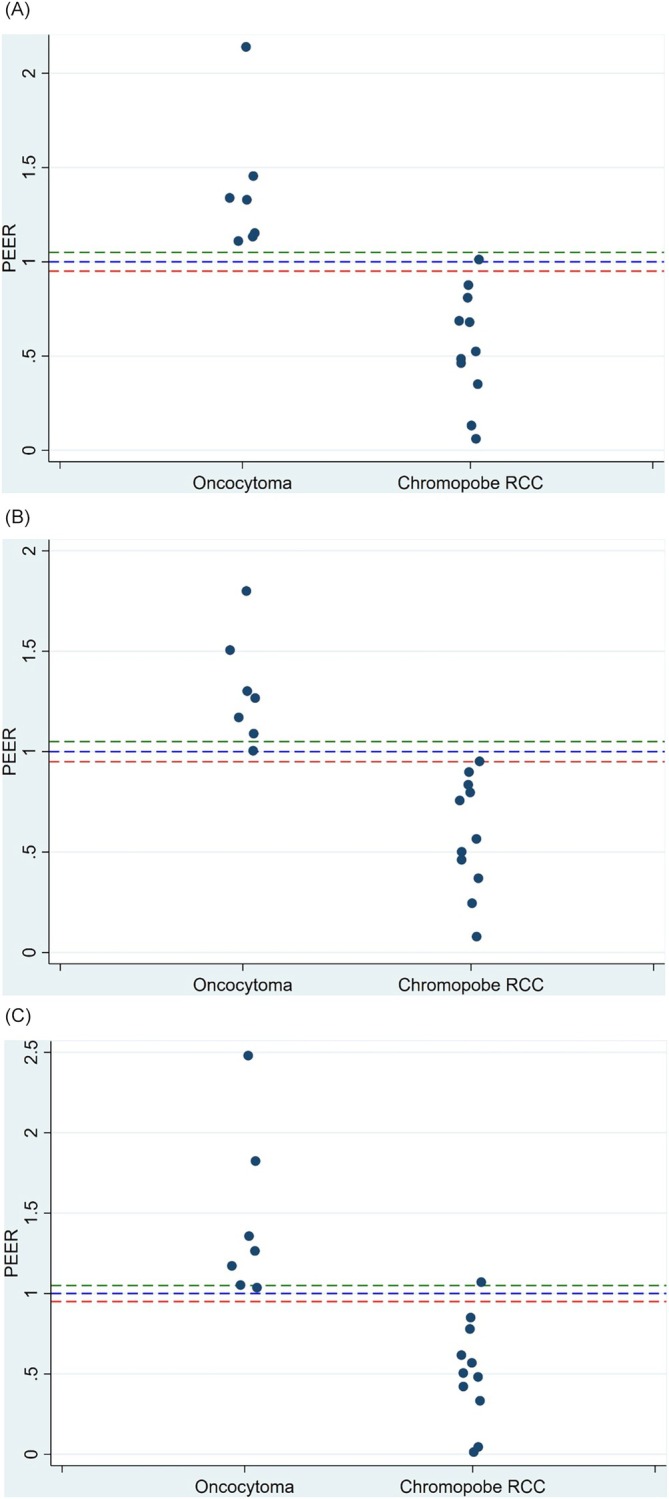
Scatter plots for PEER values for oncocytomas and chromophobe RCCs. (a) Averaged PEER values across radiologists, (b) PEER values for radiologist #1, and (c) PEER values for radiologist #2.

Median PEER values were higher for oncocytomas (1.33, interquartile range [IQR] 1.11–1.42) than for chRCCs (0.55, IQR 0.34–0.85; p < 0.001). This trend was seen for both radiologists (Table 2). The calculated correlation coefficient was high (Pearson's: 0.90), indicative of strong inter‐interpreter agreement. Scatter plots depict the averaged PEER values between the two radiologists for each oncocytoma and chRCC lesion, as well as the values obtained by the individual radiologists for each lesion (Figure 3).

**TABLE 2:** PEER measurements by two blinded radiologists stratified by the presence of oncocytoma or chromophobe RCC at surgical pathology.

	Overall	Oncocytoma	Chromophobe RCC	*p*
**N**	18	7	11	
**Median PEER (IQR)**				
**Overall** ^a^	0.89 (0.52–1.17)	1.33 (1.11–1.42)	0.55 (0.34–0.85)	<0.001
**Radiologist #1**	0.91 (0.53–1.15)	1.28 (1.06–1.51)	0.59 (0.36–0.88)	<0.001
**Radiologist #2**	0.87 (0.50–1.17)	1.24 (1.05–1.78)	0.51 (0.33–0.83)	<0.001

Abbreviations: IQR, interquartile range; PEER, peak early‐phase enhancement ratio; RCC, renal cell carcinoma.

a Analysis of dataset of averaged PEER values between the two radiologists for each tumour.

The diagnostic performance characteristics of PEER cutoff values ranging from 0.98 to 1.05 were evaluated based on visual inspection of the scatter plots (Table 3). Preference was given to maximizing sensitivity and NPV for identifying chRCC. The full range provided high performance based on AUC and individual parameters. A PEER cutoff value of 1.05 perfectly discriminated chRCC from oncocytoma for the averaged PEER measurements (Figure 3, Table 3). Using an averaged PEER value of ≤1.05 to predict the presence of chRCC resulted in 100% accuracy in pathology prediction for the averaged radiologists' measurements, with sensitivity, specificity, PPV, NPV and AUC of 100% (Table 3). For reference, radiographic tumour size alone which was larger for chRCC than oncocytoma (p = 0.04) led to an AUC of 72.1%.

**TABLE 3:** Diagnostic performance parameters for various PEER cutoffs in the overall sample and for each radiologist to differentiate chromophobe RCC from oncocytoma as diagnosed at surgical pathology.

Sample	PEER cutoff	Sensitivity	Specificity	PPV	NPV	LR+	LR‐	AUC
**Overall** ^a^								
	**0.98**	90.9%	100.0%	100.0%	87.5%	‐	0.091	95.5%
	**1.00**	90.9%	100.0%	100.0%	87.5%	‐	0.091	95.5%
	**1.05**	100.0%	100.0%	100.0%	100.0%	‐	‐	100.0%
**Radiologist #1**								
	**0.98**	100.0%	100.0%	100.0%	100.0%	‐	‐	100.0%
	**1.00**	100.0%	85.7%	91.7%	100.0%	7.0	‐	92.9%
	**1.05**	100.0%	85.7%	91.7%	100.0%	7.0	‐	92.9%
**Radiologist #2**								
	**0.98**	90.9%	100.0%	100.0%	87.5%	‐	0.091	95.5%
	**1.00**	90.9%	100.0%	100.0%	87.5%	‐	0.091	95.5%
	**1.05**	90.9%	85.7%	90.9%	85.7%	6.4	0.106	88.3%

Abbreviations: AUC, area under the curve; LR+, positive likelihood ratio; LR−, negative likelihood ratio; NPV, negative predictive value; PEER, peak early‐phase enhancement ratio; PPV, positive predictive value.

^a^ Analysis of dataset of averaged PEER values between the two radiologists for each tumour.

For radiologist #1, a PEER cutoff value of 1.05 resulted in a sensitivity of 100%, specificity of 85.7%, PPV of 91.7% and NPV of 100% for the identification of chRCC. For radiologist #2, a PEER cutoff value of 1.05 resulted in sensitivity of 90.9%, specificity of 85.7%, PPV of 90.9% and NPV of 85.7%. PEER cutoffs ranging from 0.98 to 1.05 provided high accuracy across radiologists (Table 3).

## 4 DISCUSSION

Oncocytomas are the most commonly resected benign renal masses, in part because biopsy of oncocytomas is generally inadequate to definitively exclude malignancy.^8^ Percutaneous biopsy often yields a diagnosis of “oncocytic neoplasm”, for which the differential consists of oncocytomas, chRCCs and tumours with hybrid features along a spectrum between them. Although all of these oncocytic neoplasms are less aggressive than clear cell or papillary RCC, chRCC is a malignancy with metastatic potential that warrants resection under certain circumstances,^12^ while oncocytomas are benign. Previous studies have shown that imaging evaluation using PEER measurements obtained on contrast‐enhanced CT can differentiate chRCC from oncocytoma with high accuracy.^9^, ^10^ Those studies proposed measuring PEER for tumours with biopsy pathology of oncocytic neoplasm in order to determine whether a tumour likely represents chRCC or benign oncocytoma, and thus guide management. However, the performance of PEER based on MRI images has not been previously reported.

Evaluating oncocytic tumours with MRI rather than CT may be preferred for multiple reasons. Confident non‐operative exclusion of malignancy is particularly beneficial for patients with pre‐existing poor renal function, for whom the further loss of function from resection for a benign tumour could lead to progressive chronic kidney disease. Consequently, renal insufficiency is a well‐established indication for renal mass biopsy.^13^ Complementary renal mass imaging using MRI with gadolinium‐based intravenous contrast is likely to be preferable to CT for many of these patients, as iodinated intravenous contrast used in CT is relatively contraindicated in the setting of severe renal insufficiency due to the risk of contrast‐induced acute kidney injury.^14^ Additionally, some patients may have their renal tumour initially evaluated with multiphasic MRI rather than multiphasic CT due to institutional preference or incidental reasons. Therefore, the ability to use PEER measurements on MRI examinations to increase the accuracy of non‐operative diagnosis of these renal masses could benefit a significant subset of patients.

In the present study, PEER measurements obtained using contrast‐enhanced MRI rather than CT were feasible to calculate and distinguish chRCC from oncocytoma with high accuracy. An averaged MRI PEER cutoff value of 1.05 resulted in an overall sensitivity, specificity, PPV and NPV of 100% for the detection of chRCC. This high performance is similar to the performance of CT PEER in differentiating chRCC from oncocytoma, with previously reported sensitivity of 93.2%, specificity of 87.5%, PPV of 91.1% and NPV of 90.3% regardless of CD117 status^10^ and accuracy up to 100% when applied to oncocytic tumours with known CD117 + status.^9^ A PEER cutoff value that favours high sensitivity and NPV for chRCC is preferred, in order to avoid inappropriate management of malignant lesions. This study is limited by a small sample size, and larger studies are needed to verify the optimized MRI PEER cutoff value to distinguish chRCC from oncocytoma, however, no prior studies have reported on PEER for MRI. We identified a range of values from 0.98 to 1.05 as ideal to study in additional independent cohorts. Clinically, this range could serve as an indeterminate status based on the current data and sample size, despite the perfect performance of 1.05 based on averaged values.

**FIGURE 4:**
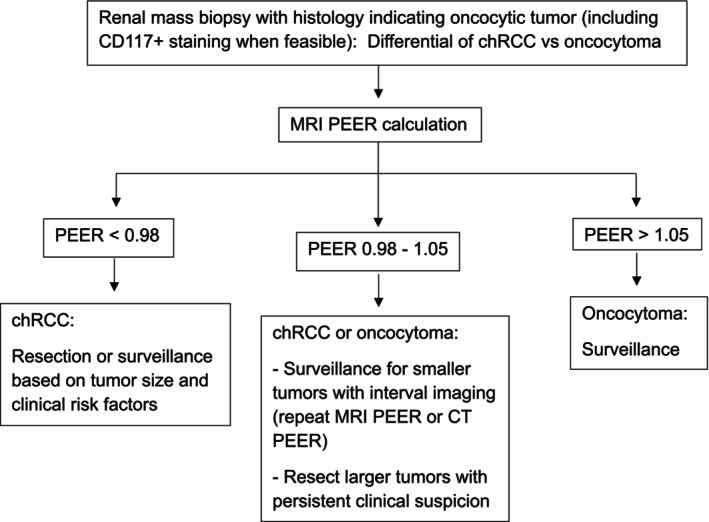
Proposed management algorithm for a patient with an oncocytic tumour based on renal mass biopsy. Upon identification of a renal lesion, percutaneous renal mass biopsy is appropriate for some patients. Histopathology evaluation of the biopsy sample (which may include CD117 staining) can result in a pathologic diagnosis of “oncocytic tumour”, for which the main differential is chRCC or oncocytoma (CD117 positive or unknown status). In this setting, PEER measurement on multiphase MRI can be used to guide management. A PEER value < 0.98 indicates the tumour is likely a chRCC and should be managed as such, either with resection or surveillance as appropriate based on factors such as tumour size and the patient's clinical status. A PEER value > 1.05 indicates an oncocytoma, which can be managed with surveillance. Tumours with PEER value ranging from 0.98 to 1.05 may be considered indeterminate, and management with resection or surveillance including repeat PEER measurement on follow‐up can be undertaken based on tumour size and clinical factors.

We propose an algorithm indicating how PEER could be used in the setting of oncocytic tumour on renal mass biopsy to guide management and avoid resection for benign oncocytomas (Figure 4). The algorithm begins with the identification of an oncocytic tumour on renal mass biopsy, due to positive CD117 staining or other histologic features limiting the differential to an oncocytic neoplasm. PEER can then be measured using multiphasic MRI to determine whether the tumour likely represents a chRCC, an oncocytoma, or remains indeterminate with both tumour types still within the differential. Management strategies for chRCC, oncocytoma and indeterminate oncocytic neoplasms based on PEER assessment are suggested, including recommendations for active surveillance if PEER assessment indicates oncocytoma.

In this study, chRCC tumours were larger than oncocytomas with a *p* value of 0.04. A similar trend was seen with prior larger studies evaluating CT PEER. However, PEER was shown to outperform size in discriminating between the tumour types in those studies [9.10]. Additionally, PEER maintained high accuracy in a previous study subgroup analysis limiting evaluation of PEER performance to discrimination of tumours measuring < 4 cm, in which there was not a statically significant difference in the size of evaluated chRCCs and oncocytomas.^10^ Nonetheless, the association of larger tumour size with chRCC may be reasonable to take into consideration as an ancillary feature when determining the management of tumours with PEER measurement in the indeterminate range, as suggested in the proposed management algorithm (Figure 4).

The cohort evaluated in this study was comprised of tumours with positive CD117 staining or unknown CD117 status on pathology. Positive CD117 staining is a histology feature that generally limits the differential diagnosis to oncocytic tumours, typically chRCC or oncocytoma, which characteristically express this marker.^8^ Previous studies have shown that PEER measured on CT best distinguishes chRCC from oncocytoma in patients with CD117 + tumours,^9^, ^10^ although high accuracy of CT PEER was also seen in a cohort which included tumours with unknown and CD117‐ status.^10^ As none of the tumours identified in this study were CD117‐, the performance of MRI PEER in cohorts including CD117‐ oncocytic tumours is not assessed. It has been recently suggested that CD117‐ oncocytic tumours may represent an indolent entity that is separate from oncocytoma and chRCC (provisionally termed “low‐grade oncocytic neoplasm”), though further evaluation is needed to more completely characterize this potential entity.^15^ Thus, MRI PEER evaluation may be most relevant for guiding the management of CD117 + oncocytic tumours, but further evaluation is needed to determine whether it is useful for guiding the management of CD117‐ oncocytic tumours.

Hybrid oncocytoma‐chromophobe tumours are also within the differential for CD117 + tumours. These lesions demonstrate borderline or mixed features of oncocytoma and chRCC, though a strict definition of hybrid tumours has not been established.^8^ Nevertheless, hybrid tumours overall demonstrate low malignant potential and may be managed similarly to oncocytomas.^16^, ^17^ However, these tumours are rare^17^ and not specifically evaluated in the current study.

The retrospective study design is a limitation which could introduce selection bias, despite blinding the radiologists measuring PEER to the pathology diagnoses. Prospective analysis of both MRI PEER and CT PEER measurements for patients with known oncocytic neoplasm status and CD117 tumour staining on renal mass biopsy would be ideal, but this is limited as many patients may not proceed to nephrectomy for a gold standard reference for comparison. Therefore, the present study design may be the most practical to replicate.

Additional study limitations include the single institution design. Also, the patient inclusion period spanned several years, increasing the likelihood of variations in patient imaging technique. However, the variety in MRI scanners and acquisition protocols utilized may reflect expected variations in imaging techniques between and within medical intuitions. Greater absolute and relative enhancement of oncocytomas compared with chRCCs has been demonstrated over multiple post‐contrast phases. Analysis of larger cohorts using CT PEER measurements showed highly accurate discrimination of chRCC from oncocytoma when using nephrographic imaging if corticomedullary images were not available, though slightly greater differentiation of the tumours was seen with the use of corticomedullary images only.^9^ Therefore, the effect of minor differences in the timing of post‐contrast imaging on the performance of PEER is expected to be small, though larger studies are needed to verify similar findings for MRI PEER.

## 5 CONCLUSION

In the present study, utilizing PEER measurements obtained from contrast‐enhanced MRI is feasible and shows promise in accurately differentiating chRCC from oncocytoma across cutoff values of 0.98 to 1.05, similar to the accuracy previously reported for PEER measurements using contrast‐enhanced CT. Larger studies are needed to further evaluate the optimal cutoff value for MRI‐based PEER. Using MRI PEER in combination with a renal mass biopsy indicating an oncocytic neoplasm may increase confidence in identifying patients with benign oncocytoma rather than chRCC, which could decrease the number of nephrectomies for benign oncocytomas.

## AUTHOR CONTRIBUTIONS

**Deanna Thorson:** Investigation, image review, manuscript drafting, review and editing. **Davide Bova:** Investigation, imaging review, supervision, manuscript review and editing. **Maria M. Picken:** Investigation, pathology review, manuscript review and editing. **Marcus L. Quek:** Study conception and design, supervision, investigation, manuscript review and editing. **Gopal N. Gupta:** Study conception and design, supervision, investigation, manuscript review and editing. **Hiten D. Patel:** Study conception and design, data management, statistical analysis, supervision, investigation, manuscript drafting, review and editing.

## CONFLICT OF INTEREST STATEMENT

Dr. Patel is supported by the Rising Tide Foundation for Clinical Cancer Research to study renal mass biopsy and a Prostate Cancer Foundation Young Investigator Award. The authors have no other relevant financial or non‐financial interests to disclose. This research did not receive any specific grant from funding agencies in the public, commercial, or not‐for‐profit sectors.
